# The relationship between perceived competence and self-esteem among novice nurses – a cross-sectional study

**DOI:** 10.1080/07853890.2022.2032820

**Published:** 2022-02-08

**Authors:** Lena Serafin, Zuzanna Strząska-Kliś, Gilbert Kolbe, Paulina Brzozowska, Iwona Szwed, Aleksandra Ostrowska, Bożena Czarkowska-Pączek

**Affiliations:** a Department of Clinical Nursing, Health Science Faculty, Medical University of Warsaw, Warsaw, Poland; b Doctoral School Medical University of Warsaw, Warsaw, Poland;; c Department of Geriatric Nurse, Health Science Faculty, Medical University of Warsaw, Warsaw

**Keywords:** Nurse education, nurse practitioners, professional development, novice nurses

## Abstract

**Background:**

Novice nurses’ responsibilities are greater than what their actual level of competence can cope with. This can cause increased levels of stress, which many studies have shown is a factor resulting in reduced self-esteem, which affects not only the well-being of nurses but also the quality of care provided.

**Aims:**

To investigate the relationship between the self-assessment of nursing competencies and self-esteem among novice nurses and the moderation role of the sociodemographic variables and intention to leave the nursing profession on this relationship.

**Material and methods:**

A correlational cross-sectional study was performed using an online questionnaire. The study was conducted between July and October 2019 among 122 novice nurses. The study tool consisted of the Rosenberg Self-Esteem Scale, the Nurse Professional Competence Scale-Short Version, and metrics. Calculations were performed using SPSS Statistics, version 25. To approach research questions hierarchical multiple regression was performed.

**Results:**

The self-esteem level of novice nurses have been identified as low. Novice nurses who declared their willingness to leave their profession had a higher level of self-esteem than nurses who did not declare this willingness. The highest-rated competencies were in the fields of nursing care and value-based nursing care, while the lowest were in the areas of development, leadership, and the organisation of nursing care. Correlations between postgraduate education and competencies in the majority subscales were revealed. The results showed a negative correlation between self-esteem and all subscales of the competence scale. Seniority and postgraduate education were important moderators in the relationship between some competence subscales and self-esteem.

**Conclusion:**

Novice nurses present a low level of self-esteem. Nurses with a higher competence level showed lower self-esteem. Developing competencies at the beginning of one’s nursing practice, which is crucial for patients’ outcomes, should be accompanied by the strengthening of novice nurses’ self-esteem.

**KEY MESSAGES**
- Novice nurses with a higher level of self-esteem more often declared their willingness to leave their profession.
- The results showed a negative correlation between self-esteem and all subscales of the competence scale.
- Seniority and postgraduate education are important moderators in the relationship between some competence subscales and self-esteem.

## Introduction

Moving to the role of a working nurse and being responsible for the lives and health of patients is often associated with a sense of confusion, uncertainty, and stress [1]. The moment of transition from being a nursing student to engaging in professional practice is a complex process, often called the struggle to develop self-esteem, including professional self-esteem [2]. This process is often accompanied by confusion and stress [1], which could negatively influence self-esteem [3–6].

Self-esteem can be defined as negative or positive beliefs about oneself; it is also an important personal resource for fighting stress and maintaining health [7]. People with high self-esteem evaluate themselves positively and are proud of their achievements, while people with low self-esteem experience feelings of worthlessness and a lack of confidence [8]. Self-esteem has been identified as a protective factor against psychological distress among nurses [9,10], is positively correlated with nurses’ well-being [11], and is related to job satisfaction through both direct and indirect effects [12]. Workers with high self-esteem reported significantly higher job satisfaction [6] and showed higher motivation levels to work, which led to higher levels of job performance [13]. On the other hand, professionals with low self-esteem are more susceptible to burnout [14–16] revealed that nurses working for over one year in the profession have a sense of increased self-esteem and competence. Professional competency is defined as the ability to use a set of knowledge, skills, and behaviours to successfully perform jobs, roles, or responsibilities [17]. Competence is also defined as a progressive experience that can be measured in five stages: beginner, advanced beginner, competent, proficient, and specialist [18]. Professional development possibilities result from specific individual features, interests, ambitions, and worldviews as well as one’s own motivation and readiness to supplement knowledge, thereby leading to the development of additional competences [19]. There is evidence that the existence of adequate competences in nurses leads to the improvement of nursing care, are crucial to establishing patient care outcomes and to increased satisfaction among the patients who receive that care [20–22]. A low level of competence in nurses may lead them to feel frustrated, dissatisfied with their work, and exhausted, which is correlated with nurses’ turnover intention through affective commitment [23,24].

The problem of nursing shortage has been highlighted as one of the biggest challenges to impact the effectiveness of health care systems [25]. In Buchan *et al.* [26] analysis of 10 countries the nurses leaving profession rate was 5–17% [26]. Furthermore, the intention to leave among novice nurses was also confirmed in previous studies [27,28] and they have been shown to leave the profession at a higher rate than any other group of nurses [29]. For both issues intention to leave the job and intention to leave the profession poor work environment characterised by factors such as understaffing and duties inconsistent with the competences have been revealed as a trigger [30,31]. Therefore, organisations’ priorities should include taking care of novice nurses by monitoring their adaptation process, minimising stress related to their new professional roles, and providing a positive environment where they can develop their competencies.

According to a systematic review by [32], newly graduated nurses’ responsibilities are greater than what their actual level of competence can cope with. Additionally, they are expected to take on the same responsibilities and duties as experienced nurses [17]. This can cause increased levels of stress, which many studies have shown is a factor resulting in reduced self-esteem. However, there is a knowledge gap in nursing research regarding this issue. To the best of our knowledge, the relationship between competencies and self-esteem has not been explored. The overall aim of this study was to assess the relationship between the self-assessment of nursing competencies and self-esteem among novice nurses. The specific aims were: (1) analysis of the self-esteem of nurses starting professional practice, (2) analysis of the relationship between sociodemographic variables and intention to leave the nursing profession and their self-esteem, (3) analysis of the level of competence and preparation for work in the profession of nurses who are starting professional practice, (4) analysis of the relationship between sociodemographic variables and intention to leave nursing profession and competence level, and (5) analysis of the moderation role of the sociodemographic variables and intention to leave nursing profession on the relationship between self-esteem and self-assessment of nursing competences.

## Material and methods

### Design

A correlational cross-sectional study was performed using an online questionnaire. The STROBE reporting guidelines were used in both the framing and reporting of this study.

### Sample/participants

A convenience sample was used for recruiting purposes. The inclusion criterion was: being a novice nurse, i.e. working as a nurse for not longer than three years based on Benner’s novice nurse definition [18], regardless of the working setting (both clinical and non-clinical experience). Exclusion criteria included: working as a nurse for over 36 months and failing to complete all the items in the questionnaire. The sample size was determined based on the rule of thumb according to Green’s procedures used to determine regression sample sizes [33,34]. An N value that was adequate for the purposes of our study was used *N* > 116. In total, 140 questionnaires were returned. Respondents with seniority of over 36 months (*n* = 11) and surveys with missing data (*n* = 7) were excluded from the analysis. A total of 122 nurses were included in the analysis.

### Data collection

The study was conducted among registered nurses between July and October 2019. A link to the survey was shared through social media, including in Polish language specialised Facebook groups.

#### Instrument

The study tool consists of three parts: the Rosenberg Self-Esteem Scale (RSES), the Nurse Professional Competence Scale-Short Version (NPCS-SF), and metrics with sociodemographic and work-characteristic data that are specific to the present study.

- The Rosenberg Self-Esteem Scale has been used for purposes of self-esteem assessment. It is a widely used self-report instrument for evaluating individual self-esteem and was developed in 1965 by Morris Rosenberg [35]. The scale is a 10-item Likert scale with items answered on a four-point scale, from strongly agree to strongly disagree, scored as follows: strongly agree—4 points; agree—3 points; disagree—2 points; and strongly disagree—1 point. Items 3, 5, 8, 9, and 10 should be reversed during scoring. The scale ranges from 10 to 40. Higher scores indicate higher self-esteem. The scale generally has high reliability. Cronbach's alpha for various samples is in the range of 0.77–0.88 [36].
- A short version of the Nurse Professional Competence Scale was used for measuring nurses' self-reported competence. The questionnaire contains 35 items with response alternatives on a seven-point scale ranging from 1 (‘to a very low degree’) to 7 (‘to a very high degree’). The NPCS-SF was developed in 2018 by Nilsson et al. to be used for purposes of measuring self-reported competence among nursing students at the point of graduation and among professional nurses in six subscales (nursing care—5 items; value-based nursing care—5 items; medical and technical care—6 items; care pedagogics—5 items; documentation and administration of nursing care—8 items; and development, leadership, and organisation of nursing care—6 items). Reliability was measured as internal consistency for the six identified factors and ranged from 0.71 to 0.86 [37].
- The third part of the questionnaire consists of sociodemographic and work-related variables such as age, sex, seniority, educational level, postgraduate education, characteristics of a place of work, intention to change one’s workplace, and intention to leave the profession. This part has been developed by the authors of the study and has been used to characterise the study group.

### Ethical considerations

The study was approved by the Ethical Board at the Medical University of Warsaw (reference number AKBE/285/2019). Each participant received a cover letter explaining the study purpose and the terms of participation. The cover letter also ensured confidentiality. The respondents’ names were not recorded on the questionnaire, thus rendering the data anonymous. Informed consent was indicated by voluntary participation in the survey. The act of sending the completed questionnaire was synonymous with consent to participate in the study.

### Data analysis

Calculations were performed using SPSS Statistics, version 25 (IBM Corp.). The normality of the data distribution was evaluated using the Kolmogorov-Smirnov test. In the first step, the relationship between the NPCS-SF and RSES dimensions were calculated through the use of correlation analysis (Pearson correlation coefficient). The differences between the two groups of quantitative variables were compared using the non-parametric *U* Mann-Whitney test (sexes and intention to leave the current workplace and intention to leave the profession).

The relationship between NPCS-SF, RSES and education level and postgraduate education was determined through the use of Spearman’s rank correlation coefficient; and seniority through the use of the Pearson correlation coefficient; and place of work through the use of the analysis of variance (ANOVA).

In the next step, moderation analyses were performed using A. Hayes's PROCESS macro. Sociodemographic variables such as seniority, level of education, postgraduate education, workplace, intention to leave one’s current job, and intention to leave the profession were introduced as moderators for hierarchical multiple regression analysis. *α* ≤ 0.05 was adopted as the level of significance for the analyses.

### Rigor/validity

Our study used the Polish adaptation of the Rosenberg Self-Esteem Scale of [38]. The validation of the Polish version of RSES revealed high reliability, with a Cronbach’s alpha of 0.81–0.83, and confirmed content validity. The Nurse Professional Competence Scale had not previously been used in Poland. Therefore, a translation process following [39] guidelines was conducted.

In June 2019, we conducted a pilot study among 22 novice nurses. Face validity revealed no need for changes in the survey. Additionally, this analysis confirmed the high reliability of the chosen tools. For the Rosenberg Self-Esteem Scale, Cronbach's alpha was 0.93. For the subscales of NPCS-SF, Cronbach's alpha was as follows: nursing care—0.70, value-based nursing care—0.85, medical and technical care—0.78, care pedagogics—0.94, documentation and administration of nursing care and development—0.75, and leadership and organisation of nursing care—0.87.

## Results

### Sample characteristics

The inclusion criterion was fulfilled by 122 respondents (116 women and 6 men). Therefore, we firstly checked on whether there were any differences between these groups. The results of the *U* Mann-Whitney test revealed significant differences between men and women in terms of value-based nursing care (*Z* = −2.10; *p* = .036) and medical and technical care (*Z* = −1.99, *p* = .047). Men obtained higher scores for both scales. Due to the small number of respondents in the group of men, as well as to statistically significant differences, men were excluded from further analysis.

Sample characteristics are presented in Table 1. The majority of respondents had a bachelor’s degree in nursing and continuing education, had taken between one and three postgraduate courses, they worked mainly in a clinical setting. Intention to leave one’s current workplace was declared by 58.6% of respondents, while the intention to leave the nursing profession was declared by 31.9% of respondents.

**Table 1.** Sample characteristics.

	*N* (%)	Mean (SD) min-max
**Age**		24.06 (1.67)
		**22–56**
**Seniority (months)**		16.9 (10.09)
		**1–36**
**Education**		
BSN	5 (4.3)	
BSN + continuing study	70 (60.3)	
MSN	41 (35.3)	
**Number of undertaken postgraduate courses**		
0	31 (26.7)	
1–3	61 (52.6)	
> 3	24 (20.7)	
**Place of work**		
Operating room/anestesiology	30 (25.9)	
Surgical ward	42 (36.2)	
General care ward	28 (24.1)	
Primary healthcare	13 (11.2)	
Other	3 (2.6)	
**Intend to leave current workplace**		
No	48 (41.4)	
Yes	68 (58.6)	
**Intend to leave nursing profession**		
No	79 (68.1)	
Yes	37 (31.9)	

SD: standard deviation; BSN: Bachelor of Nursing Science; MSN: Master of Nursing Science; min: minimum; max: maximum.

### Novice nurses’ self-esteem

The self-esteem of novice nurses measured by RSES was rated at 19.15 points, which means that they present their self-esteem slightly below half on a scale of 10–40 points (Table 2). Analysis of the comparison of two groups showed differences between the self-esteem level of nurses who wanted to leave the profession and nurses who didn’t (*Z* = −2.53, *p* = .011, *r* = 0.23). Novice nurses who declared their willingness to leave their profession had a higher level of self-esteem than nurses who did not declare their willingness to leave the profession. Otherwise, there was no relationship between the level of self-esteem and the level of seniority, education, postgraduate education, workplace, or willingness to leave one’s current workplace.

**Table 2.** Pearson correlation coefficients between NPCS-SF and RSES dimensions.

	M	SD	1	2	3	4	5	6	7
Self-esteem	19.15	5.42	1						
Nursing care	85.22	85.71	−0.42**	1					
Value-based nursing care	86.33	87.14	−0.35**	0.80**	1				
Medical and technical care	80.71	83.33	−0.28**	0.73**	0.71**	1			
Care pedagogics	80.57	85.71	−0.26**	0.68**	0.76**	0.80**	1		
Documentation and administration of nursing care	84.48	85.71	−0.33**	0.76**	0.78**	0.82**	0.78**	1	
Development, leadership and organisation of nursing care	72.52	76.19	−0.19*	0.60**	0.60**	0.78**	0.75**	0.73**	1

M: mean; SD: standard deviation.

*p < .05; **p < .01.

### Novice nurses’ competences

The highest-rated competencies measured by NPCS-SF were in the fields of nursing care and value-based nursing care, while the lowest-rated competencies were in the areas of development, leadership, and organisation of nursing care. Mean values for all measured subscales are presented in Table 2. Analysis of the relationship between sociodemographic factors and measured competencies showed positive correlations between postgraduate education and competencies in the following subscales: nursing care (*r* = 0.271, *p* = .001), medical and technical care (*r* = 0.319, *p* < .001), care pedagogics (*r* = 0.268, *p* = .001), documentation and administration of nursing care (*r* = 0.256, *p* = .002), and development, leadership, and organisation of nursing care (*r* = 0.293, *p* < .001). In addition, there was no relationship or group differences in NPCS-SF due to the level of seniority, education, workplace, and willingness to leave the workplace and profession.

### Relationship between novice nurses’ self-esteem and competences

A negative correlation was revealed between self-esteem and all subscales of the NPCS-SF, which means that as competence increases, self-esteem decreases. The strongest negative relationship was between nursing care and self-esteem (*r* = −0.417, *p* < .001) and value-based nursing care and self-esteem (*r* = −0.345, *p* < .001). The results of the correlation are presented in Table 2.

### Seniority and postgraduate education as moderators of the relationship between self-esteem and competences

To determine whether some sociodemographic variables are significant moderators of the relationship between the RSES and NPCS-SF dimensions, several multiple linear regression analyses were performed using the PROCESS macro by A. Hayes. The method of grand mean centred predictors was used for the analysis. Detailed analysis results are discussed below.

**Figure 1.**
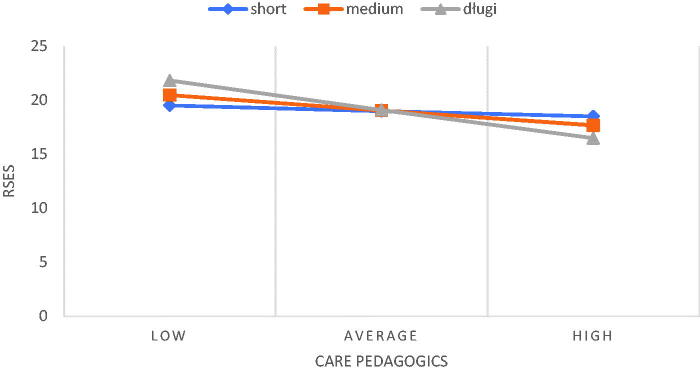
The relationship between documentation and administration of nursing care and RSES including the moderating role of seniority.

The hierarchical regression analysis with the interactive component showed that seniority was an important moderator between the documentation and administration of nursing care and RSES (Table 3; Figure 1). The moderation effect was significant for nurses with medium (*B* = −0.27; *p* = .001, 95%CI [−0.43; −0.12]) and long work experiences (*B* = −0.44; *p* < .001, 95%CI [−0.64; −0.23]). For people with less work experience (below 1 SD from the average), the relationship between this dimension of NPCS-SF and RSES was insignificant.

**Table 3.** Regression coefficients and change of R2 for the interaction between NPCS-SF subscales and seniority.

*X*	*B*	*SE*	*t*	*p*	*95% CI*	*ΔR*	*F*(1,112)	*p*
Nursing care	< −0.01	<0.01	−0.98	.331	[−0.01–<0.01]	0.01	0.95	.331
Value-based nursing care	< −0.01	<0.01	−0.51	.609	[−0.01–0.01]	<0.01	0.26	.609
Medical and technical care	−0.01	<0.01	−1.56	.121	[−0.01–<0.01]	0.02	2.45	.121
Care pedagogics	−0.01	<0.01	−1.90	.060	[−0.01–<0.01]	0.03	3.61	.060
Documentation and administration of nursing care	−0.01	<0.01	−2.04	.043	[−0.02 – < −0.01]	0.03	4.18	.043
Development, leadership and organisation of nursing care	−0.01	<0.01	−1.65	.103	[−0.01–0.01]	0.02	2.71	.103

X: independent variable – NPCS-SF subscales; B: unstandardised regression coefficient; SE: standard error; t: *T*-Test result; *p*: level of significance; Δ*R*^2^: R2 parameter change for the model after enabling interaction; F: ANOVA value for the model change; *p*: level of significance for the change.

**Figure 2.**
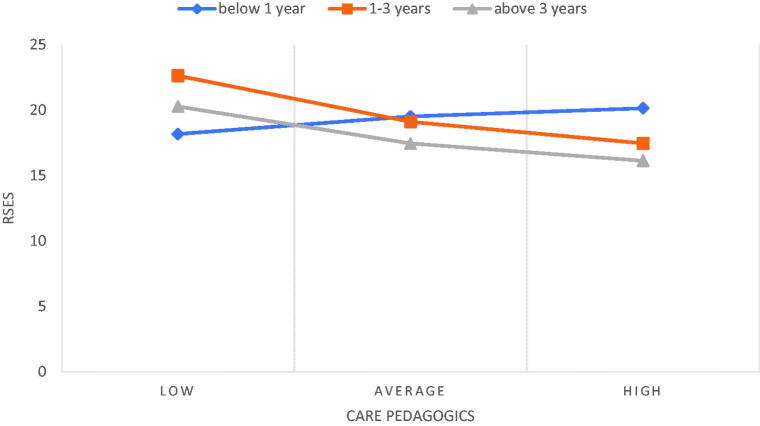
The relationship between care pedagogics and RSES including the moderating role of postgraduate education.

**Figure 3.**
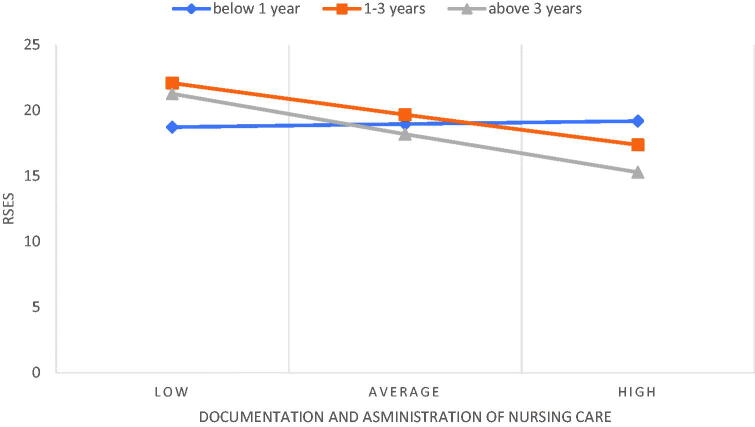
The relationship between documentation and administration of nursing care and RSES including the moderating role of postgraduate education.

Dummy coding was used to analyse the assessment of postgraduate education as a moderator in the relationship between the NPCS-SF and RSES dimensions, with people who do not take any courses serving as a reference category, in accordance with the coding (Table 4). The analysis showed two significant interactions between care pedagogics and participation in postgraduate courses, as well as between the documentation and administration of nursing care and participation in postgraduate courses 1 to 3 times after starting work (Table 4). The moderation effect turned out to be significant for nurses who participated in postgraduate courses between 1 to 3 times after starting work (*B* = −0.50; *p* ≤ .001, 95%CI [−0.78; −0.23]) and for those who participated in postgraduate courses > 3 times (*B* = −0.40; *p* = .045, 95%CI [−0.80; −0.01]) (Figure 2). For people who have not participated in any courses, the relationship between care pedagogics and RSES was not significant. Moreover, the moderation effect of postgraduation education on the relationship between the documentation and administration of nursing care and RSES was revealed as significant for nurses who participated in postgraduate courses between 1 to 3 times after starting work (*B* = −0.38; *p* ≤ .001, 95%CI [−058; −0.19]) and for those who participated in postgraduate courses > 3 times (*B* = −0.48; *p* = .025, 95% CI [−0.90; −0.06]) (Figure 3). For people who have not participated in any courses, the relationship between documentation and administration and RSES was not significant. In addition, the education of nurses, the workplace, the intention to leave one’s current job, and the intention to leave the profession were not moderators of the relationship between the dimensions of NPCS-SF and RSES.

**Table 4.** Regression coefficients and change of R2 for the interaction between NPCS-SF subscales and postgraduate courses.

*X*	*B*	*SE*	*t*	*p*	*95% CI*	*ΔR^2^*	*F*(2,110)	*p*
Nursing care								
int1	−0.18	0.09	−1.89	.062	[−0.37–0.01]	0.03	2.04	.135
int2	−0.23	0.15	−1.51	.134	[−0.52–0.07]			
Value-based nursing care								
int1	−0.20	0.11	−1.82	.071	[−0.46–0.02]	0.03	1.71	.185
int2	−0.19	0.14	−1.33	.185	[−0.46–0.09]			
Medical and technical care								
int1	−0.18	0.09	−1.95	.053	[−0.35–<0.01]	0.03	1.97	.144
int2	−0.15	0.11	−1.39	.178	[−0.37–0.07]			
**Care pedagogics**								
**int1**	**−0.24**	**0.08**	**−2.94**	**.004**	**[−0.41- −0.08]**	**0.07**	**4.52**	**.013**
**int2**	**−0.21**	**0.10**	**−2.16**	**.033**	**[−0.40- −0.02]**			
**Documentation and administration of nursing care**								
**int1**	**−0.25**	**0.10**	**−2.28**	**.025**	**[**−**0.44 –** −**0.03]**	0.05	3.05	.052
int2	−0.29	0.15	−1.96	.051	[−0.58–0.01]			
Development. leadership and organisation of nursing care								
int1	−0.12	0.07	−1.71	.091	[−0.27–0.02]	0.03	1.90	.155
int2	−0.14	0.08	−1.75	.083	[−0.29–0.02]			

X: independent variable – NPCS-SF subscales; B: unstandardised regression coefficient; SE: standard error; t: *T*-Test result; *p*: level of significance; Δ*R*^2^: R2 parameter change for the model after enabling interaction; F: ANOVA value for the model change; *p*: level of significance for the change; W1: 1: people taking courses 1–3 times; 0: other people; W2: 1: persons taking courses> 3 times; 0: other people; Int1: X × W1; Int2: X × W2.

## Discussion

Self-esteem in nursing is described as an essential part of both personal and professional identities [40,41]. An integral part of becoming a nurse is forming self-esteem, which shapes the knowledge, attitudes, and skills necessary for the nursing profession [42]. Therefore, our analysis focussed on the assessment of self-esteem and competence among nurses starting their professional practice and on the exploration of the relationship between these variables.

The average self-esteem level presented by the respondents in our study (19.15 points) was identified as lower than that in other studies conducted among nurses [9,11,43] and even among nursing students [3,44,45]. Although there are no set breakpoints for RSES, a score between 20 and 30 points is considered a normal range; a result less than the norm is an indicator of low self-esteem [43]. The moment of transition from being a nursing student to engaging in professional practice is a complex process, often called the struggle to develop self-esteem, including professional self-esteem [2]. Therefore, taking care of novice nurses and increasing their self-esteem is important, as it prevents numerous problems defined by self-esteem low level; these problems, which are broadly described in the literature, include low job involvement, higher risk of stress, mental health problems, low job satisfaction and burnout [5,6,15,46,47,48]. In the study conducted by [49], several factors had a positive relationship to new graduate nurse confidence in interprofessional collaboration: the availability and accessibility of the manager and educator, the number of different disciplines worked with daily, the number of team strategies, and satisfaction with the team. Therefore, starting a career in a supportive work environment with the commitment of managers and co-workers is crucial in developing novice nurses’ self-esteem.

Our study has shown that novice nurses with a higher level of self-esteem more often declared their willingness to leave their profession. This, considered in the broader context of the relationship between self-esteem and competencies, indicates that they represent also a group of people with a lower competence level. The negative relationship between competence and nurses’ turnover intention was earlier revealed [24,50], but transition programs are strong evidence to support newly qualified nurses’ retention [51]. However, new graduate nurses feel reduced levels of support over time [29]. Therefore, fostering positive relationships with the supervisor and colleagues, improving working and employment conditions, and developing competence must be carried out continuously.

Value-based nursing care and nursing care have been indicated as being the best-developed competencies, while development, leadership, and the organisation of nursing care were rated the least-developed competencies. Nursing care is the main domain of nursing education, which is confirmed by the novice nurses’ declaration regarding the best preparation in this area. In addition, value-based nursing care is a conscious and prudent approach to providing care. Positive experiences with providing a good quality of care have proven to be important for newly graduated nurses’ competence and their desire to continue in the nursing profession [52]. The effective preparation of newly graduated nurses in the field of ethics was also confirmed by [53]. A majority of the respondents in our study had continued their master's studies and completed postgraduate education, which may also confirm the results of previous studies in which nurses pursuing a bachelor’s degree or higher were generally found to have higher awareness and application of the nurses’ professional values in practice than did nurses with lower levels of nursing education [54]. Moreover, there is evidence that nurses' professional values increased along with their perception of caring quality, and as their working experience at the current clinic increased [55,56]; this confirms that nurses develop professional values through a variety of psychological and educational processes [57]. The weakest preparation in the area of the development, leadership, and organisation of nursing care should be carefully considered. Earlier studies [58,59] have indicated the need for stronger leadership skills at the point when nursing education begins. Leadership education depends on expert input from both academic and practice-based educators. In addition to introducing leadership theory and competence, students must be exposed to positive leadership practices during clinical placements to facilitate theory-practice integration [59]. The process of educating nurses is complex and each of its elements complements the competencies necessary to provide high-quality care. However, when analysing the most frequently indicated weaknesses of newly graduated nurses and the current challenges of health care systems, more attention should be paid to developing competencies such as leadership and organisation of nursing care during pre- and postgraduate training.

In our study, nurses' competencies in most areas increased as per the completed level of postgraduate education; a majority of the respondents undertook additional postgraduate education. It is important to note that in the early years of practice, nurses’ competencies increase significantly, both through their active participation in education and through the experience of independent work. Importantly, newly graduated nurses need more support in the areas of competence development [60] and wellbeing at work [60,61], as they present a high-stress level [62]. Moreover [52], revealed that newly graduated nurses’ higher general competence indicates a stronger overall occupational commitment. Undeniably, competence is a critical attribute for safe, ethical, and high-quality care [62]. Therefore, developing nurses’ competencies and monitoring this process should be a matter not only for individuals but also for healthcare organisations in the context of the system’s functioning.

Nevertheless, our study has shown that the self-esteem of young nurses decreases as their level of competence increases. This presents a new finding, although the relationship is very logical. Following Socrates' ancient paradigm, "I know I know nothing," the development of shared competencies can cause a broadening of the perspective of knowledge, which can ultimately lead to a decrease in self-esteem. However, taking into account the fact that nurses must continually develop competencies, and considering the great importance of self-esteem in fostering effective and satisfying work among nurses (which, beyond the benefits for the individual, also has an impact on the functioning of entire organisations), a deeper analysis of the described phenomenon should be provided. In our study, greater seniority increases the negative relationship between the level of documentation and administration of nursing care and self-esteem. However, it has also been shown that the higher number of postgraduate courses increases the negative relationship between the level of documentation and administration of nursing care and care pedagogics and self-esteem. It seems that with the formal improvement of competencies (postgraduate courses), as well as the increasing experience that novice nurses gain at work, their self-esteem decreases; this may be the result of a mismatch between the ordered duties and the actual level of nurses’ competence, which increases very quickly in the first years of professional practice. Institutional activities, such as the proper organisation of work patterns, have a huge impact on the utilisation level of competence and the efficiency of workers. Full utilisation of employee competence is possible only with adequate organisational solutions [63]. Therefore, Competence-Based Management (CBM), i.e. linking a competence model to required competencies to perform the jobs within an organisation, should be implemented in nursing practice. Previous studies have shown many positive CBM effects, such as higher employee motivation, increased employee retention, a higher level of job satisfaction, a higher quality of health care, and higher patient safety and patient satisfaction [65], which are consistent with today's goals for nursing care around the world. Nevertheless, this new finding (i.e. the negative relationship between competencies and self-esteem) requires further research, including longitudinal investigations.

## Limitations

Although this study has used meticulous methods, some limitations necessitate cautious interpretation of the findings. First, this study used a cross-sectional design, which allowed us to describe the phenomenon of the negative correlation between competencies and self-esteem. However, this made it impossible for us to present the analysis of results over time. The results of this study can be used as a basis for further research; a longitudinal design could be used to track changes over time and implemented interventions The presented results require a deeper analysis of a new finding, which would create a better understanding of our outcomes and identify when and what supportive activities are appropriate. Additionally, dissemination of the survey through social media could influence the characteristics of the study group in terms of their level of self-esteem. Moreover, while the size of the study group was adequate based on the chosen calculation method, the number was at the low end of the acceptable limit. With that in mind, a larger sample containing more equal representation in terms of sex would allow for a comparison between groups, which has been revealed in our study as statistically significant. In addition, data regarding the work locations of participants would improve the sample characteristics.

## Conclusion

In conclusion, our present study revealed a negative correlation between the competencies and self-esteem of novice nurses. Novice nurses present an alarmingly low level of self-esteem. An analysis of the negative consequences that this creates for both the individual and the organisation indicates a need to strengthen novice nurses’ self-esteem. Strengthening their self-esteem and developing competencies should be the domain of undergraduate training. Then, management based on supporting nurses’ competence development should incorporate evaluating their fast improvement, especially at the beginning of professional practice. A lack of attention to this aspect could be a reason employees are underestimated, which may decrease their level of self-esteem. Moreover, the lack of employee competence development, which results in a disproportionately high level of self-esteem, increases the desire of novice nurses to leave the profession, which is now an area of focus among healthcare organisations around the world. Therefore, a solution worth verifying in future interventions that support this issue is the implementation of competence-based management in nursing.

## Data Availability

The data that support the findings of this study are available from the corresponding author, [LS], upon reasonable request.
